# A Novel Biclustering Approach to Association Rule Mining for Predicting HIV-1–Human Protein Interactions

**DOI:** 10.1371/journal.pone.0032289

**Published:** 2012-04-23

**Authors:** Anirban Mukhopadhyay, Ujjwal Maulik, Sanghamitra Bandyopadhyay

**Affiliations:** 1 Department of Computer Science and Engineering, University of Kalyani, Kalyani, West Bengal, India; 2 Department of Computer Science and Engineering, Jadavpur University, Kolkata, West Bengal, India; 3 Machine Intelligence Unit, Indian Statistical Institute, Kolkata, West Bengal, India; Semmelweis University, Hungary

## Abstract

Identification of potential viral-host protein interactions is a vital and useful approach towards development of new drugs targeting those interactions. In recent days, computational tools are being utilized for predicting viral-host interactions. Recently a database containing records of experimentally validated interactions between a set of HIV-1 proteins and a set of human proteins has been published. The problem of predicting new interactions based on this database is usually posed as a classification problem. However, posing the problem as a classification one suffers from the lack of biologically validated negative interactions. Therefore it will be beneficial to use the existing database for predicting new viral-host interactions without the need of negative samples. Motivated by this, in this article, the HIV-1–human protein interaction database has been analyzed using association rule mining. The main objective is to identify a set of association rules both among the HIV-1 proteins and among the human proteins, and use these rules for predicting new interactions. In this regard, a novel association rule mining technique based on biclustering has been proposed for discovering frequent closed itemsets followed by the association rules from the adjacency matrix of the HIV-1–human interaction network. Novel HIV-1–human interactions have been predicted based on the discovered association rules and tested for biological significance. For validation of the predicted new interactions, gene ontology-based and pathway-based studies have been performed. These studies show that the human proteins which are predicted to interact with a particular viral protein share many common biological activities. Moreover, literature survey has been used for validation purpose to identify some predicted interactions that are already validated experimentally but not present in the database. Comparison with other prediction methods is also discussed.

## Introduction

Interactions between proteins are important biochemical reactions which determine different biological processes. Analysis of the regulation between viral and host proteins in different organisms is an important step to uncover the underlying mechanism of various viral diseases. Human immunodeficiency virus (HIV) is a lentivirus (a member of the retrovirus family with long incubation period) that can lead to acquired immunodeficiency syndrome (AIDS), a condition in humans in which the immune system begins to fail, leading to life-threatening infections [1]. HIV-1 is a species of the HIV virus that relies on human host cell proteins in virtually every phase of its life cycle. One of the main goals in research of Protein-Protein Interaction (PPI) is to predict possible viral-host interactions. This is specifically aimed at assisting drug developers targeting protein interactions for the development of specially designed small molecules to inhibit potential HIV-1–human PPIs. Targeting protein-protein interactions has relatively recently been established to be a promising alternative to the conventional approach to drug design [2], [3].

There are several computational approaches for predicting PPIs [4]. In [5], different data sources have been combined using Bayes classifier for predicting PPIs in yeast. Different classification methods have been compared in [6] and it has been found that random forest classifier performs the best. In a study by Yamanishi *et al.*, new protein interactions have been predicted using a variant of kernel canonical correlation analysis [7]. In [8], a decision tree has been constructed to predict co-complexed protein pairs by integration of genomic and proteomic data. Afterwards, some kernel methods have also been described for predicting novel PPIs [9]. An approach called Mixture-of-Feature-Experts (mixture of classifiers) is proposed in [10] to predict the set of interacting proteins in yeast and human cells. In this approach the features are split into roughly homogeneous sets of feature experts (classifiers). Each of the individual experts uses logistic regression and finally their scores are combined using another logistic regression.

Most of the above approaches are mainly used for determining PPIs in a single organism, such as yeast, human etc. However, determination of PPIs across multiple organisms such as between viral proteins and the corresponding host proteins can contribute to the development of new therapeutic approaches and design of drugs for these viral diseases. There are some recent computational approaches in predicting and analyzing HIV-1-human PPIs. Tastan et al. [11] proposed a classification model based on random forest classifier for predicting new HIV-1-human PPIs. The authors extended their method by integrating a semi-supervised approach for selecting positive interactions in [12]. Doolittle et al. recently proposed a structural similarity based approach for predicting HIV-1–human protein interactions [13]. A support vector machine classifier based approach is presented in [14]. In a recent study by MacPherson et al. [15], an analysis of the HIV-1–human PPI network using a biclustering method has been done to identify significant host-cellular subsystems. A similar approach is adapted in [16] to find immunodeficiency gateway proteins and their involvement in microRNA regulation. In [17], a preliminary study on association rule mining based prediction of HIV-human PPI has been reported.

The computational methods for predicting HIV-1–human PPIs are mainly based on designing some classifiers which need both positive and negative samples for PPIs. Although there are several online resources that systematically store information about experimentally validated interacting proteins, there is no such resource for non-interacting proteins which should be used as the ideal negative samples. Therefore in most of the works in this area, negative samples are prepared by taking random protein pairs which are not found in the interaction database. This is done with the expectation that this random protein pairs are less likely to interact physically, which may not be true always. The performance of the classifier highly depends on the choice of the negative samples.

With this observation, in this article we have proposed an approach based on association rule mining (ARM) [18] that uses information of positive samples of experimentally validated PPIs only [19], [20]. The PPI information among HIV-1 and human proteins are organized as a binary matrix with rows representing the human proteins and columns representing the HIV-1 proteins or vice-versa. Thereafter, novel association rule mining technique based on a biclustering method has been developed and the proposed technique is used for discovering association rules among the viral proteins as well as human proteins. Finally these rules have been utilized to predict some new viral-host interactions and their biological relevance has been studied.

## Results and Discussion

In this section, the procedure for mining ARs from HIV-1–human PPI network has been described. First we describe the preparation of the input data set. Thereafter, how to apply the proposed algorithm on the input data set to discover highly confident rules and how these rules are used to predict new interactions are discussed. Finally, the results of mining ARMs from HIV-1-human PPI network are reported and discussed.

### Preparation of the Input Data Set

The HIV-1–human PPI database [19] consists of total 2534 interactions between 19 HIV-1 proteins and 1432 human proteins. We have constructed a binary matrix of human and viral proteins, 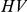 of size 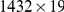 (**File S1**), and its transpose matrix 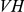 of size 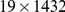 (**File S2**). An entry of 1 in the matrices denotes the presence of interaction between the corresponding pair of human and HIV-1 proteins, and an entry of 0 represents the absence of any information regarding the interaction of the corresponding human and viral proteins. Initially it is treated as non-interaction. The resulting binary matrices is treated as the input to the BiMax biclustering algorithm [21].

### Finding ARs from the PPI Data Set

As discussed above, the rows of the input binary matrix 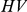 represent the human proteins and the columns represent the viral proteins. Here each row (human protein) has been considered as a transaction and each column (viral protein) has been considered as an item. Therefore, in each transaction, the items, for which the corresponding value in the matrix is 1, are considered to be purchased by the transaction. This means, with a human protein, some of the viral proteins (for which corresponding entry in the row is 1) interact. Thereafter, the proposed ARM algorithm is applied on these transactions to find highly confident ARs. As an example, a discovered AR may be of the form:
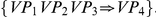
Here 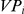
s, 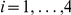, represent four HIV-1 proteins. In words, the rule can be interpreted as follows: If the HIV-1 proteins 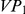, 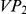 and 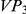 interact with some human protein, the HIV-1 protein 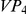 is also likely to interact with the same human protein. Corresponding to each rule, there is an associated set of human proteins for which the rule is true.

In a similar fashion, we also extract the ARs from the 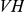 matrix, where the viral proteins are in the rows and the human proteins are in the columns. Hence from this matrix, the extracted rules are of the following form:
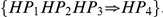
Here 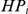
s, 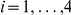, represent four human proteins. In this case also, there is an associated set of viral proteins for each such rule.

#### Filtering extracted ARs

Here we have used a two-step filtering process to obtain high-confident, non-redundant and most general ARs. The two steps are as follows:

#### Step-1: Removing Less-confident rules

In this step, the rules that have confidence less than the provided 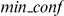 value are removed.

#### Step-2: Removing redundant rules

For removing the redundant rules, the following filtration is adopted: Consider a rule 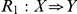. We say that the rule 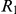 is redundant if there exists another rule 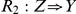 with same consequent, and 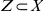 while both 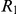 and 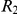 have confidence greater than or equal to 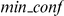. In this case the rule 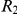 is said to be more general than rule 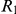. We remove the rules that are redundant.

The two steps of the filtering process are applied one by one on the initially extracted rules to obtain high-confident, non-redundant and most general rules for further use.

#### Prediction of new interactions from ARs

**Figure 1:**
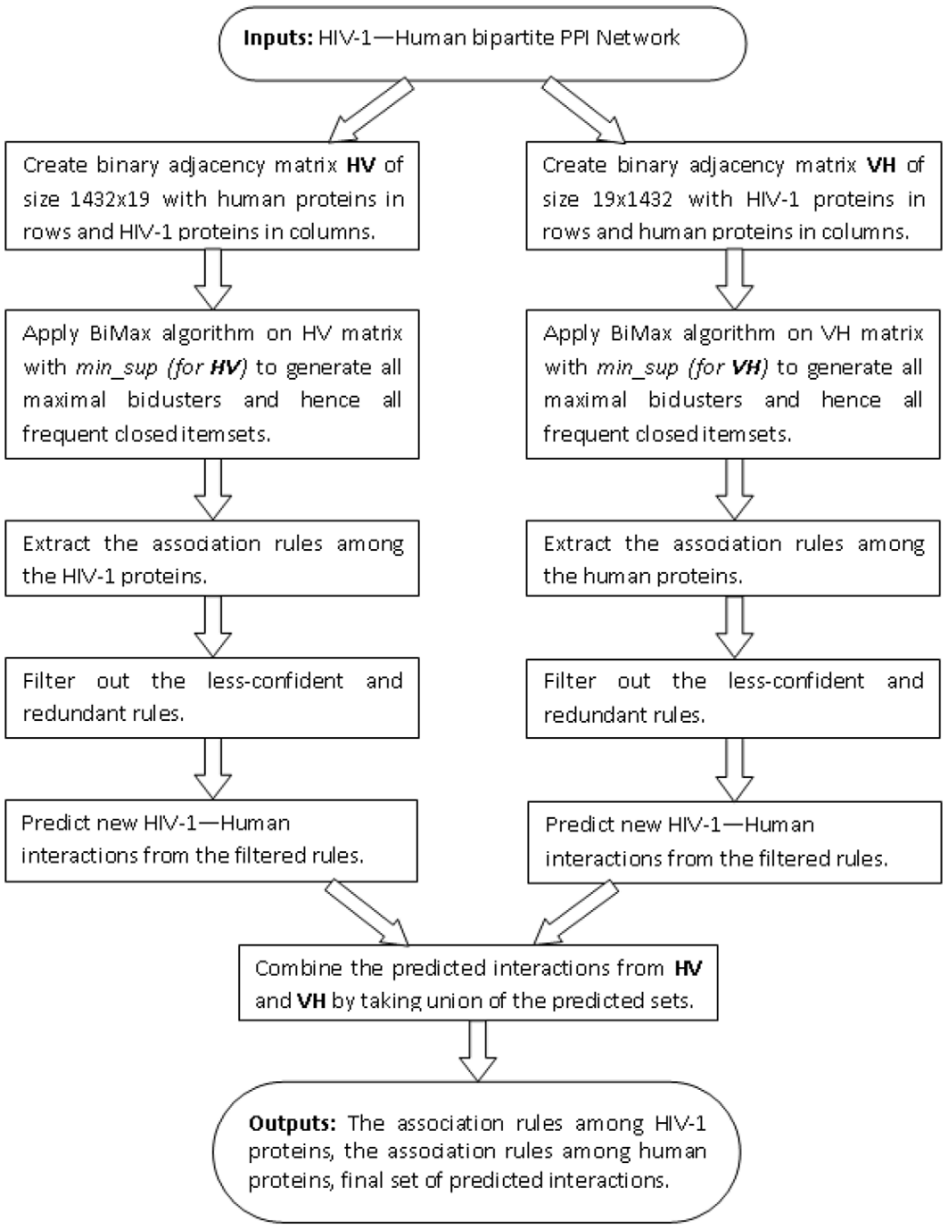
Flowchart of the process of predicting new interactions.

We have utilized the filtered ARs to predict new viral-host interactions as follows: Consider again the rule 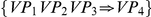 obtained from the 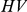 matrix. Suppose in the frequent itemsets, the antecedent of the rule is true for 8 human proteins 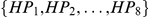. Now without loss of generality, assume that among these 8 human proteins, the consequent of the rule is true for the first 6 human proteins 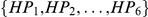. Therefore the rule has a confidence of 75% (6 out of 8), which can be thought as reasonably high. From this we can predict that the viral protein 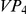 is also likely to interact with the human proteins 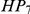 and 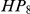 and the confidence of this prediction is 75%. Thus two new interactions are predicted (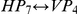 and 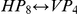. The same procedure can be applied on the rules obtained among the human proteins for the 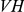 matrix to predict some new interactions with certain confidence levels. This way, from all the high-confident non-redundant rules, we predict some new interactions with certain levels of confidence from both 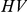 and 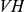 matrices and the union of these sets of predicted interactions is used as the final predicted set of interactions. Fig. 1 shows the flow chart of the procedure used in this article for predicting new interactions.

The following text describes the experimental results. Note that we have two binary matrices in hand: 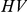 and 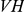. As there are 19 HIV-1 proteins and 1432 human proteins, the sizes of the 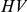 and 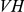 matrices are 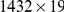 and 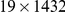, respectively. These two matrices are processed separately and finally the predicted interactions are merged to get the final predictions.

### Results on 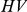 Matrix

As the HIV-1 proteins are in the columns of 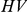 matrix, we obtain the rules among HIV-1 proteins from this. Since the PPI data set is very sparse, the minimum support value should be low enough to obtain sufficient number of frequent closed itemsets. After several experiments, the 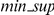 value is set to 20 (∼1.4%) and the 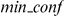 value is set to 70%.

**Figure 2:**
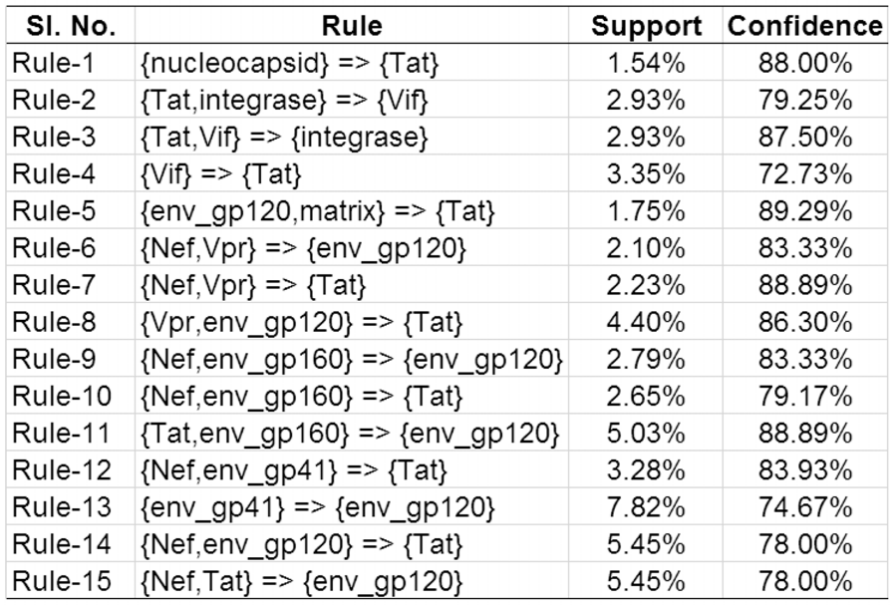
Final Set of 15 Association Rules among HIV-1 Proteins Generated from HV Matrix.

By applying the BiMax algorithm with the specified parameters discussed above, we extracted 48 all-1 maximal biclusters, i.e., 48 frequent closed itemsets. From these frequent closed itemsets, 123 unique rules with single consequent are generated. Thereafter, we apply the filters on the extracted rules. After applying the Step-1 and Step-2 of the filtration process, the number of rules reduces to 26 and 15, respectively. Fig. 2 shows the final set of 15 rules among the viral proteins.

Subsequently we apply the proposed method to predict new interactions. When the rules shown in Fig. 2 are applied on the 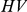 matrix, it generates a total of unique 140 new predicted interactions with different confidence levels. As can be seen from the Fig. 2, there are 4 different HIV-1 proteins (env_gp120 (5 rules), integrase (1 rule), Tat (8 rules) and Vif (1 rule)) in the consequent parts of the rules, hence all the predicted interactions involve only these HIV-1 proteins. The number of unique human proteins involved in these interactions is 130.

### Results on 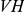 Matrix

For the 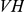 matrix, there are large number of items (1432 human proteins) in the columns of the matrix, whereas the number of rows of the matrix is only 19 (corresponding to 19 viral proteins). Hence, in this case the minimum support value can be much larger than that in case of 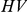 matrix. Experimentally, we set the 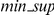 value to 5 (∼26.32%) and the 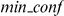 value to 70%.

**Figure 3:**
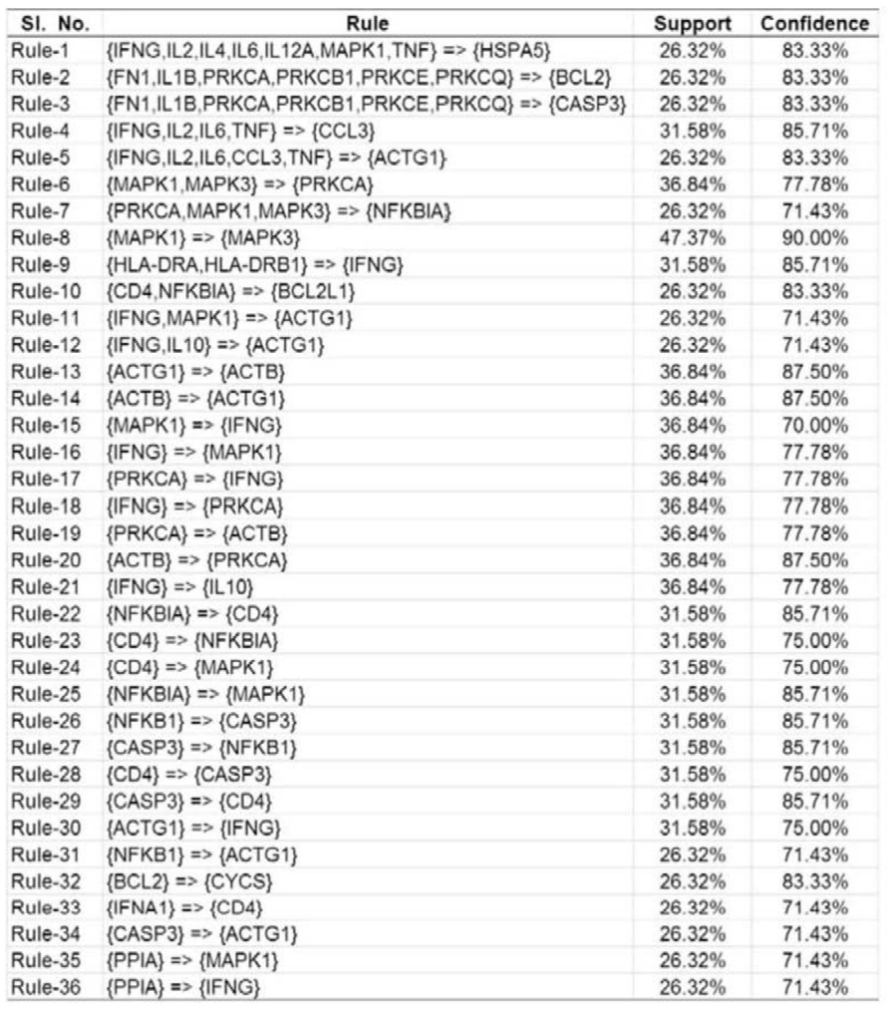
Final Set of 36 Association Rules among HIV-1 Proteins Generated from VH Matrix.

Application of BiMax algorithm with the above parameters yields 74 all-1 maximal biclusters, which is equivalent to 74 frequent closed itemsets. Thereafter we extracted 361 unique rules with single consequent from these frequent closed itemsets. Subsequently, the extracted rules are filtered and the Step-1 and Step-2 of the filtration process minimize the number of rules to 50 and 36, respectively. In Fig. 3, we have shown the final set of 36 rules among the human proteins.

Next, the proposed method is applied to predict new interactions. The rules shown in Fig. 3 in turn predict 43 new interactions from the 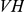 matrix. As is evident from Fig. 3, there are 16 different human proteins (ACTB (2 rules), ACTG1 (6 rules), BCL2 (1 rule), BCL2L1 (1 rule), CASP3 (3 rules), CCL3 (1 rule), CD4 (3 rules), CYCS (1 rules), HSPA5 (1 rule), IFNG (5 rules), IL10 (1 rule), MAPK1 (4 rules), MAPK3 (1 rule), NFKB1 (1 rule), NFKBIA (2 rules), PRKCA(3 rules)) in the consequent parts of the rules. Therefore all the predicted interactions from 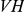 matrix involve these human proteins. On the other hand, the number of unique HIV-1 proteins in these interactions is 15 (capsid, env_gp120, env_gp160, env_gp41, Gag_Pr55, matrix, Nef, nucleocapsid, p6, retropepsin, Rev, RT, Vif, Vpr, Vpr).

### Predicted Interactions

**Figure 4:**
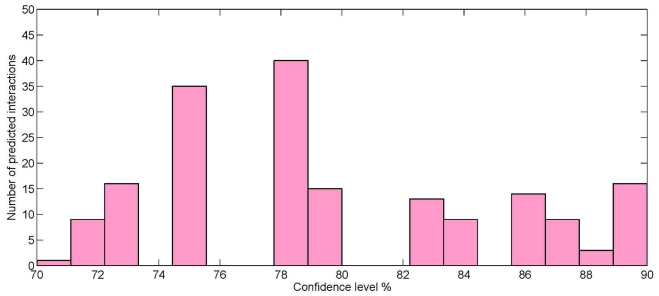
Distribution of the number of predicted interactions at different confidence levels.

The predicted interactions from 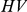 matrix (140 interactions) and 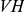 matrix (43 interactions) are merged together by taking union. Only three interactions have been found to be common in both the sets (env_gp120
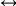
ACTB, env_gp120
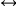
ACTG1 and env_gp120
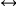
BCL2L1). Hence it is evident that the predictions from 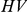 and 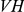 matrices are mostly different from each other. This indicates that both forms of the adjacency matrix are equally important in predicting new interactions. Taking the union of the two predicted sets, the final set of predictions containing 180 unique interactions are formed. We studied the distribution of the confidence levels of the interactions. In this regard, Fig. 4 shows the histogram of the distribution of the number of predicted interactions at different confidence level. It can noted from the figure that at confidence level 78%–80%, there are the maximum number (40) of predicted interactions. There are also many interactions with high confidence value (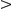
80%).

This final set of 180 predicted interactions involves 17 unique HIV-1 proteins (capsid, env_gp120, env_gp160, env_gp41, Tat, integrase, Gag_Pr55, matrix, Nef, nucleocapsid, p6, retropepsin, Rev, RT, Vif, Vpr, Vpu) and 140 human proteins. It is stated in [20] that some HIV-1 proteins interact with multiple proteins belonging to specific protein complex or cellular pathway, which represent specific cellular activities. Conversely, 37% of the host proteins in the database interacts with more than one HIV-1 protein. In the predicted set, we found that some HIV-1 proteins interact with multiple human proteins and biological relevance study based on gene ontology reveals that the human proteins interacting with a single viral protein share common biological activities and participate in common cellular pathways. Moreover, it is also observed that in the predicted interactions, many human proteins (14.29%) interact with more than one HIV-1 proteins similar to the observation reported in [20].

**Figure 5:**
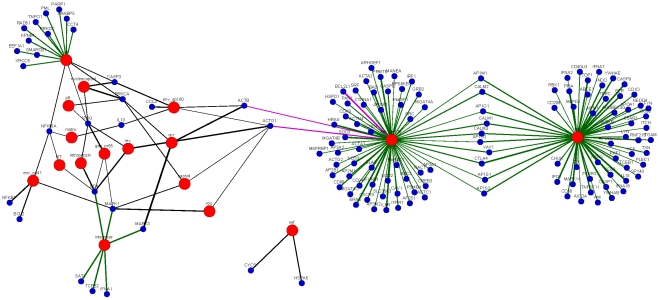
The predicted bipartite network involving 17 HIV-1 proteins and 140 human proteins. HIV-1 proteins are represented by large red circles and human proteins are represented by small blue circles. The interactions predicted from VH matrix are represented by black lines and the interactions predicted from HV matrix are represented by green lines. Line widths are proportional to the confidence of the predictions.

For the purpose of illustration, the predicted bipartite network has been shown in Fig. 5. It appears from the figure that there are mainly two viral hub proteins, env_gp120 and Tat that interact with 64 (35.56%) and 59 (32.78%) human proteins, respectively. Interestingly, in the original interaction database it is found that env_gp120 and Tat interact with 33% and 30% host proteins [20]. Hence these percentages are very near to those in the predicted interaction data set. The HIV-1 protein env_gp120 is embedded in the HIV envelop which helps the virus to attach to and fuse with the target cell, whereas Tat mainly plays the role of increasing the rate of transcription in the host cell. So both of them play important role in viral life cycle and are expected to interact with many proteins in the host cell for possible infection. The next HIV-1 hub protein in the predicted network is Vif, which is predicted to interact with 15 human proteins. Vif protein is known to be responsible for disruption of antiviral activities of human cells and expectedly interacts with many human proteins for this purpose. The degrees of other viral proteins in the predicted network are as follows: capsid - 3, env_gp160 - 5, env_gp41 - 4, integrase - 6, Gag_Pr55 - 3, matrix - 2, Nef - 2, nucleocapsid - 3, p6 - 2, retropepsin - 2, Rev - 3, RT - 2, Vpr - 3, and Vpu - 2. Interestingly, Nef is predicted to interact with two human proteins CYCS and HSPA5, with which, no the viral protein is found to interact in the predicted set. The complete set of predicted interactions is given in the additional File **S3** along with the predictions from both 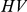 and 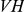 matrices separately.

### Biological Relevance of Predicted Interactions

In this section, we examine the properties of human proteins that interact with each HIV-1 proteins based on gene ontology based study. The results of these experiments are reported below.

#### Interactions with env_gp120

As stated above, 64 human proteins have been predicted to interact with the viral protein env_gp120. Here we attempt to study the biological relationships among these 64 human proteins. First, we conducted a gene ontology (GO) based study to find whether there are any significant GO terms in the three categories of GO using DAVID functional annotation tool (http://david.abcc.ncifcrf.gov). To obtain the significant non-redundant GO terms, we have utilized a recently developed web server REVIGO (http://revigo.irb.hr/) [22] which takes as input a list of GO terms along with p-values and a GO-based semantic similarity measure, and outputs the set of non-redundant terms along with *Dispensability* values for each term. Lower value of *Dispensability* indicates lesser redundancy of the corresponding term [22]. We have used the default parameter setting of REVIGO as provided in the web server. Tables 1, 2 and 3 show the significant and non-redundant GO terms for certain thresholds of p-value and dispensability (as shown in the respective captions) under Biological Process, Cellular Component and Molecular Function Categories, respectively. It is evident from the tables that the group of human proteins that are predicted to interact with env_gp120 are biologically related and possess common biological activities. It is interesting to note from Table 1 that 21.67% of these human proteins are involved in biological process *membrane organization*. Also Table 2 shows that many of these proteins are component of *coated membrane* (20%). This is an important observation because as stated earlier env_gp120 is embedded in the HIV envelop which helps the virus to attach to and fuse with the target cell. In this process it needs to interact with many membrane proteins and our predicted human proteins seem to be largely related with membrane activities, and there is high chance that env_gp120 interacts with these proteins. From Table 1, it can also be found that some of these human proteins are involved in the biological process of *death* (18.33%) and *regulation of defense response to virus by virus* (8.33%), which are important for promoting antiviral immune response mechanism and thereby limiting viral replication. When env_gp120 possibly interacts with these human proteins they affect their activities and as a result the immune response system may fail. This indicates that interaction of env_gp120 with these human proteins may lead to cell death.

**Table 1:** Significant non-redundant GO terms (p-value
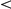
1E-03, Dispensability
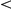
0.05) under Biological Process category found in the human proteins that are predicted to interact with HIV-1 protein env_gp120.

Sl. No.	GO-id	Term	*p-value*	% of Proteins	Dispensability
1	GO:0007568	aging	8.91E-05	10.00	0.00
2	GO:0016192	vesicle-mediated transport	8.11E-10	28.33	0.00
3	GO:0016265	death	6.87E-04	18.33	0.00
4	GO:0050690	regulation of defense response to virus by virus	1.82E-08	8.33	0.00
5	GO:0030029	actin filament-based process	4.63E-04	11.67	0.04
6	GO:0016044	membrane organization	3.82E-08	21.67	0.04
7	GO:0006928	cell motion	7.03E-04	15.00	0.04

**Table 2:** Significant non-redundant GO terms (p-value
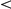
1E-08, Dispensability
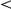
0.05) under Cellular Component category found in the human proteins that are predicted to interact with HIV-1 protein env_gp120.

Sl. No.	GO-id	Term	*p-value*	% of Proteins	Dispensability
1	GO:0030131	clathrin adaptor complex	1.61E-19	20.00	0.00
2	GO:0031982	vesicle	3.54E-11	33.33	0.00
3	GO:0012505	endomembrane system	3.83E-09	31.67	0.00
4	GO:0048475	coated membrane	2.54E-15	20.00	0.04

**Table 3:** Significant non-redundant GO terms (p-value
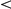
1E-02, Dispensability
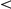
0.05) under Molecular Function category found in the human proteins that are predicted to interact with HIV-1 protein env_gp120.

Sl. No.	GO-id	Term	*p-value*	% of Proteins	Dispensability
1	GO:0008565	protein transporter activity	7.43E-08	13.33	0.00
2	GO:0008454	alpha-1,3-mannosylglycoprotein 4-beta-N-acetylglucosaminyltransferase activity	5.27E-05	5.00	0.00
3	GO:0042802	identical protein binding	1.35E-03	16.67	0.00

#### Interactions with Tat

Tat has the second most highest degree (59) in the predicted interactions, and thus needs special attention. For Tat also we have conducted similar GO study and the results are shown in Tables 4, 5, and 6. It is evident that in this case also, the human proteins have many significant terms in each of the three categories of GO, however, the significant terms are sufficiently different from that in the case of env_gp120. It appears from Table 4 that many of these human proteins are involved in *defense response*, *positive regulation of apoptosis* and *response to virus*. These are vital observations since Tat is known to vastly increase the level of transcription of HIV dsRNA, thus allowing HIV an explosive response once certain amount of Tat is produced. This endangers the immune activities inside the cell. Therefore it is expected that Tat interacts with the human proteins that are involved in immune and defense activities to make them inactive. Moreover Tat has been found to act as toxin producing cell death via apoptosis in uninfected T cells, which assists in progression towards AIDS. Furthermore, it appears from Table 6 that 9.09% proteins are associated with the molecular function *protein transporter activity*, which means these proteins may be highly involved in transporting infection signal to different parts of a cell or between cells. Hence the predicted human proteins interacting with Tat are quite intuitive since they share many biological properties related to HIV infection and progression.

**Table 4:** Significant non-redundant GO terms (p-value
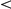
1E-03, Dispensability
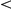
0.15) under Biological Process category found in the human proteins that are predicted to interact with HIV-1 protein Tat.

Sl. No.	GO-id	Term	*p-value*	% of Proteins	Dispensability
1	GO:0006897	endocytosis	2.23E-05	14.55	0.00
2	GO:0006952	defense response	6.46E-08	27.27	0.00
3	GO:0070661	leukocyte proliferation	6.71E-04	7.27	0.00
5	GO:0001775	cell activation	1.20E-04	14.55	0.03
6	GO:0043065	positive regulation of apoptosis	2.47E-04	16.36	0.03
7	GO:0009615	response to virus	8.62E-04	9.09	0.10

**Table 5:** Significant non-redundant GO terms (p-value
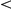
1E-02, Dispensability
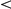
0.15) under Cellular Component category found in the human proteins that are predicted to interact with HIV-1 protein Tat.

Sl. No.	GO-id	Term	*p-value*	% of Proteins	Dispensability
1	GO:0005829	cytosol	2.30E-10	43.64	0.00
2	GO:0031982	vesicle	5.27E-05	21.82	0.00
3	GO:0048475	coated membrane	1.13E-04	9.09	0.00
4	GO:0031226	intrinsic to plasma membrane	2.47E-03	23.64	0.06

**Table 6:** Significant non-redundant GO terms (p-value
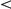
1E-02, Dispensability
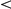
0.15) under Molecular Function category found in the human proteins that are predicted to interact with HIV-1 protein Tat.

Sl. No.	GO-id	Term	*p-value*	% of Proteins	Dispensability
1	GO:0008565	protein transporter activity	3.17E-04	9.09	0.00
2	GO:0016814	hydrolase activity, acting on carbon-nitrogen (but not peptide) bonds, in cyclic amidines	6.05E-03	5.45	0.00
3	GO:0051219	phosphoprotein binding	1.39E-04	7.27	0.00

#### Interactions with Vif

The other less-dense HIV-1 hub protein found in the predicted interactions is Vif, the virion infectivity factor. This protein is known to interfere with the immune system’s defences and increases the infectivity of the HIV particle. The 15 human proteins that are predicted to be interacting partners of Vif are found to share many common significant GO terms as depicted in Tables 7, 8, and 9. The proteins are mainly involved in nuclear and protein import activities. Also, a large number of proteins (more than 50%) are found to be involved in *regulation of apoptosis*. This indicates that these human proteins may also be in the viral infection pathway leading to cell death through apoptosis. Interestingly, we found that out of these 15 human proteins, 5 of them are involved in pathways in cancer (KEGG: hsa05200) suggesting that possible viral infection may lead to some type of cancer.

**Table 7:** Significant non-redundant GO terms (p-value <1E-04, Dispensability <0.15) under Biological Process category found in the human proteins that are predicted to interact with HIV-1 protein Vif.

Sl. No.	GO-id	Term	*p-value*	% of Proteins	Dispensability
1	GO:0000060	protein import into nucleus, translocation	2.34E-08	35.00	0.00
2	GO:0006259	DNA metabolic process	3.65E-06	50.00	0.03
3	GO:0033554	cellular response to stress	6.97E-06	50.00	0.04
4	GO:0042981	regulation of apoptosis	5.17E-05	50.00	0.04

**Table 8:** Significant non-redundant GO terms (p-value
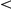
1E-02, Dispensability
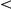
0.15) under Cellular Component category found in the human proteins that are predicted to interact with HIV-1 protein Vif.

Sl. No.	GO-id	Term	*p-value*	% of Proteins	Dispensability
1	GO:0005654	nucleoplasm	8.64E-06	57.14	0.00
2	GO:0031974	membrane-enclosed lumen	1.03E-03	57.14	0.00
3	GO:0005829	cytosol	7.62E-03	42.86	0.12

**Table 9:** Significant non-redundant GO terms (p-value <1.5E-02, Dispensability <0.15) under Molecular Function category found in the human proteins that are predicted to interact with HIV-1 protein Vif.

Sl. No.	GO-id	Term	*p-value*	% of Proteins	Dispensability
1	GO:0008139	nuclear localization sequence binding	1.94E-05	21.42	0.00
2	GO:0008134	transcription factor binding	1.30E-02	28.57	0.02

#### Interactions with other HIV-1 proteins

The other HIV-1 proteins have smaller degrees in the predicted network. We also studied the relationships among the human proteins that are predicted to interact with some common HIV-1 protein through GO and KEGG pathway enrichment test using DAVID. The HIV-1 protein capsid interacts with ACTG1, MAPK1 and PRKCA. Interestingly, these three proteins interact with each other as found in the STRING database (http://string-db.org). Another envelop protein env_gp160 is predicted to interact with 5 human partners ACTB, ACTG1, CASP3, CCL3, and IL10. These five partners have significant involvement in *cell motion*, *regulation of B-cell activation/proliferation*, and *regulation of T-cell activation/proliferation*. Thus these proteins are involved in humoral and cell-mediated immune response and viral infection affects these immune activities. The env_gp141 is predicted to interact with BCL2, MAPK1, NFKB1 and NFKBIA, which are found to be significantly involved in the *B-cell/T-cell signaling pathway*, *apoptosis* and *programmed cell death*. Moreover all of them are involved in the *signaling pathway of prostate cancer*. This is interesting since it suggests that HIV-1 infection through env_gp41 may indirectly cause prostate cancer. The Gag_Pr55 is predicted to interact with CD4, IFNG, MAPK1 and all these three proteins take part in the *positive regulation of immune system process*. The proteins are also found in the *T-cell receptor signaling pathway*. Thus they are responsible for the immunity of the host. The human proteins CD4, IFNA1, MAPK1, MAPK3, SAT1, and TCEB2 are predicted to interact with integrase. Out of these six human proteins three (MAPK1, MAPK3 and TCEB2) are found to be in the *T-cell receptor signaling pathway* and the pathway leading to *renal cell carcinoma*. The human proteins IL10, NFKBIA are predicted to interact with the viral protein matrix. Both of these human proteins actively participate in *maintenance of protein locations in cell* and *regulation of transcription factor activity*. The viral protein Nef is predicted to interact with CYCS and HSPA5. Expression of Nef causes T-cell activation and it also promotes the survival of infected cells by downmodulating the expression of several surface molecules important in host immune function. Interestingly, these two human proteins have involvement in *apoptosis* and *programmed cell death*. The HIV-1 protein nucleocapsid is found to interact with CASP3, IFNG and PRKCA, and importantly, these three human proteins are significantly involved in *negative regulation of cell proliferation*, *positive regulation of apoptosis* and *programmed cell death*. Moreover they are involved in the KEGG pathway of *natural killer cell mediated cytotoxicity (hsa04650)*. The human proteins predicted to interact with the HIV-1 proteins p6, retropepsin, Rev, RT, Vpr and Vpu also share many biological activities related to immune response, apoptosis and programmed cell death.

### Evidences from Recent Literature

To establish that the predicted interactions have potentials to exist in reality, we have extensively searched PUBMED to find recent reports on interactions between HIV-1 and human proteins which are not included in the interaction database considered here. We have found some of the predicted interactions which are experimentally validated and reported in recent literature. For example, we have predicted the interaction between env_gp120 and CASP8 with confidence 83.33% (File **S3**). In [23] it has been found that env_gp120 expression induces CASP8 activation and apoptosis. Also our prediction between env_gp120 and CD86 (confidence 83.33%) has been supported in [24], which found that env_gp120 infection causes upregulation of CD86. The human protein NOS3 is also predicted to interact with env_gp120 with confidence level 74.67% by our method. It has been shown in [25] that env_gp120 and TNF-alpha synergistically reduce NOS3 expression in both mRNA and protein level. Two other human proteins, SOD2 and SRC are predicted to interact with env_gp120 with confidence levels 88.89% and 78%, respectively. These interactions are also supported by the works in [26] and [27], respectively. Differential regulation of SOD2 in neurons and astroglia is affected by env_gp120 [26]. It is reported in [27] that soluble env_gp120 activates multiple protein kinases in primary human monocyte-derived macrophages, including the SRC family kinase. In [28], it is established that env_gp41 peptide 6358 activates the CD74-mediated ERK/MAPK pathway and significantly enhanced HIV-1 infection. It is interesting that our prediction includes the interaction between env_gp41 and MAPK1 with confidence level 77.78%. We have also predicted interaction of MAPK1 with the viral protein Gag_Pr55 with 71.43% confidence. In [29], the authors have shown that MAPK/ERK-2 interacts with the poly-proline motif present in the capsid region of Gag_Pr55. In [30], it is reported that HIV-1 Tat protein enhances RANKL (TNFSF)-mediated osteoclast differentiation. We have predicted the interaction of Tat with TNFSF with confidence level 86.30%. These evidences establish that many of our predicted interactions, which are not in the HIV-1–human interaction database, have already been reported and exist in reality. This demonstrates the utility of the proposed method.

### Overlaps with Existing Predictions

**Figure 6:**
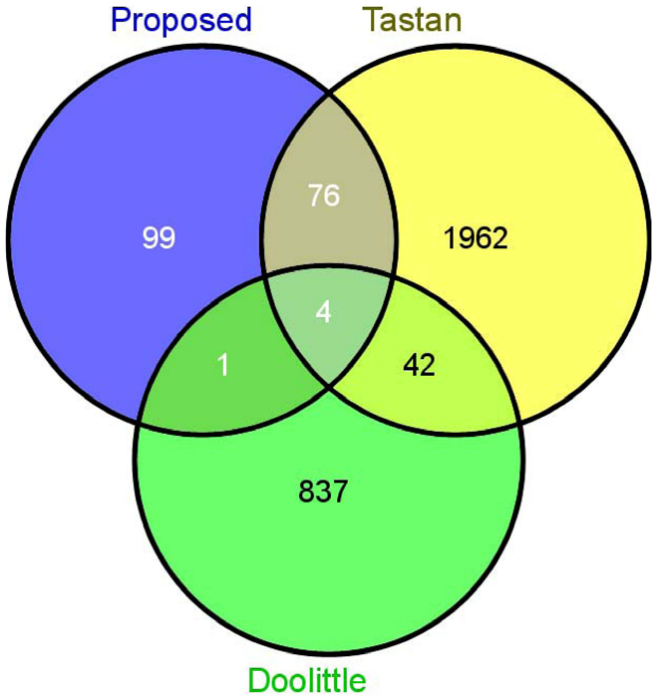
Venn diagram showing the overlaps of predicted interactions provided by the proposed method, by Tastan et al. and by Doolittle et al.

We have compared our predictions with that of Tastan et al. [11] and Doolittle et al. [13]. Tastan et al. used a random forest classifier based technique and predicted 3372 interactions. Each prediction has an associated interaction score. Out of these, 2084 interactions are predicted to be novel interactions (not in the database). The interaction scores of these interactions vary from 0 to 4.11 with a mean value of 0.5284. In [13], a structural similarity based method have been proposed for predicting protein interactions between HIV-1 and human and a total of 884 unique interactions are predicted. Fig. 6 gives the Venn diagram showing the overlaps among the three prediction studies. It is evident that the predictions from these three methods have reasonably low overlaps. This is not quite unexpected because even large-scale experimental protein interaction studies do not typically show high degree of overlaps among them. Moreover, these three prediction studies use completely different methodologies and predict different number of interactions. From Fig. 6 it can be noticed that the predictions from the present study have overlap of 80 interactions with that of Tastan et al., whereas the number of overlaps between our method and Doolittle’s method is only 5. We have reported the common predictions between the studies in this paper and Tastan et al. in File **S4**. These 80 common predictions may be closely observed and are interesting candidates for further studies. Note that among these common interactions, 4 interactions (env_gp120
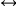
CASP8, env_gp120
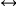
SRC, env_gp41
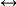
MAPK1, and Gag_Pr55
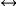
MAPK1) are already establish by experimental validation as discussed in the previous section. The 5 common interactions between our study and that of Doolittle et al. are capsid
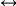
MAPK1, env_gp41
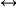
NFKB1, env_gp41
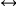
MAPK1, integrase
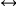
CD4, and integrase
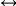
MAPK1. There are 4 interactions which are predicted by all three methods. These interactions are capsid
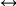
MAPK1, env_gp41
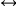
MAPK1, integrase
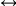
CD4, and integrase
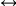
MAPK1. As these 4 interactions are common to all methods, these might be important candidates for further investigations. Among these, as discussed in the previous section, env_gp41
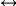
MAPK1 already has evidence in literature [28]. In summary we can say that little overlaps among the predicted interactions from different methods suggest that these methods might be complementary to each other for identifying novel interactions between HIV-1 and human proteins.

### Summary and Discussion

In this article we have posed the problem of predicting new HIV-1–human protein interactions based on the existing PPI database as an association rule mining problem. This is motivated by the difficulty in posing the problem as a classification one due to lack of proper negative PPI samples. In this regard, a novel association rule mining approach based on BiMax biclustering method has been proposed. The proposed technique has been used to mine the frequent closed itemsets from the HIV-1–human PPI database organized as a binary matrix. The non-redundant association rules of high confidence have been generated and a novel method for predicting new interactions from the generated rules has been proposed. The proposed method has been shown to predict new viral-host interactions with certain confidence levels. For validation of the predicted new interactions, gene ontology-based and pathway-based study have been performed. Ptak et al. [20] noticed that some HIV-1 proteins interact with multiple proteins belonging to specific protein complex or cellular pathway, which represent specific cellular activities. Similar trend is noticed for the predicted interactions as well. The GO and pathway studies reveal that the human proteins that are predicted to interact with a particular viral protein share many common biological activities that are mainly related to immune response, apoptosis and programmed cell death. Moreover, in some of the cases, we found that the group of human proteins interacting with a viral protein are involved in the pathway of some type of cancer suggesting that HIV-1 infection may lead to several types of cancer through its infection pathway. Moreover, we have shown some evidences from recent literature which demonstrate that some of our predicted interactions are already established through experimental validation. Finally we have discussed the overlaps of our predicted interactions with that by two other existing methods for HIV-1–human PPI prediction.

One possible criticism of the proposed technique might be that it can only predict new interactions involving the human proteins present in the HIV-1–human database. However, if some viral protein is predicted to interact with a human protein, the human proteins that are structurally similar to the target human protein or that have same domains as in the target human protein, are also likely to interact with the concerned viral protein [13]. Hence it is evident that although our method does not directly predict interactions involving human proteins not present in the interaction database, it can predict the possibility of interactions of HIV-1 proteins with new cellular factors that are structurally similar with the human proteins involved in predicted interactions.

In this work, we have used a simple model that does not differentiate among the different types of interactions present in the interaction database. The main interest of this study is to propose a novel idea of utilizing a biclustering algorithm to derive association rules and using the derived rules to predict new interactions. Hence it is not straightforward to annotate the predicted interactions. Although GO-based study gives us some insights into the sub-cellular locations of predicted interactions (GO cellular components), a more detailed study is needed and initial assumptions are to be revised for more clear overview of the viral replication process. Our future plan is to consider all possible types of interactions present in the database initially to build the interaction matrix (matrix will no longer be a binary one), and also to consider the directions of interactions (virus-to-host, host-to-virus or undirected). Starting with this, the derived rules will then be able to predict the annotated interactions along with their directions. This is expected to more clearly explain the process of HIV-1 replications by predicting interactions in different stages of viral life cycle with possible sub-cellular compartments where the interactions are taking place. The authors are now working in these directions.

## Materials and Methods

### HIV-1–Human PPI Data Set

The interaction information reported between HIV-1 and human proteins has been prepared based on a recently published PPI interaction data set [19] (http://www.ncbi.nlm.nih.gov/RefSeq/HIVInteractions/). The database was accessed in March 2011 to prepare the PPI data set for the present study. There are total 2534 interactions between 19 HIV-1 proteins and 1432 human proteins. The interaction information in the database is derived from small scale protein interactions curated from published materials. The PPIs reported in the database are specifically annotated according to the nature of the interactions including over 68 interaction types such as “binds to”, “interacts with”, “upregulates” and “phosphorylates”. The types of interactions can be broadly classified into direct physical interactions (e.g., “binds to”), that comprise of 32% of the interactions and indirect interactions (e.g., “upregulates”) that comprise of 68% of the interactions. Both direct and indirect interactions are considered in our study since indirect interactions also contain valuable information about the characteristics of the interacting proteins. Including the information, at the beginning of the analysis is expected to be beneficial for predicting novel interactions, both direct as well as indirect. Moreover, the natures of interactions are also not considered separately. We have constructed a binary matrix of human and viral proteins, 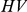 of size 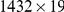, and its transpose matrix 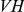 of size 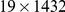. An entry of 1 in the matrices denotes the presence of interaction between the corresponding pair of human and HIV-1 proteins, and an entry of 0 represents the absence of any information regarding the interaction of the corresponding human and viral proteins. Initially it is treated as non-interaction.

### Association Rule Mining

The principle of association rule mining (ARM) lies in the market basket or transaction data analysis. Much information is hidden in the day to day transactions taking place in supermarkets. For example a customer who is buying nappy also likes to purchase baby food in the same time. Association analysis is the discovery of rules showing attribute–value associations that occur frequently [31]. Let 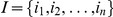 be a set of 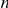 items and 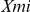 be an itemset where 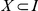. A 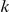-itemset is a set of 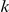 items. Let 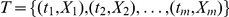 be a set of 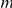 transactions, where 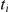 and 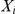, 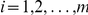, are the transaction identifier and the associated itemset respectively. The *cover* of an itemset 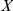 in 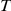 is defined as follows:
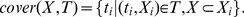
(1)The *support* of an itemset 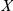 in 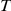 is
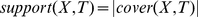
(2)and the *frequency* of an itemset is
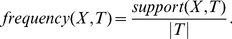
(3)Thus support of an itemset 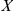 is the number of transactions where all the items in 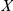 appear in each transaction. The frequency of an itemset is the probability of its occurrence in a transaction in T. An itemset is called frequent if its support in 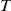 is greater than some threshold 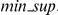 The collection of frequent itemsets with respect to a minimum support 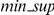 in 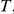 denoted by 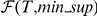 is defined as
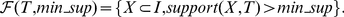
(4)The objective of ARM is to find all rules of the form 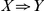, 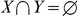 with probability 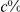, indicating that if itemset 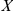 occurs in a transaction, the itemset 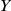 also occurs with probability 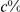. 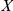 and 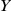 are called the antecedent and consequent of the rule respectively. Support of a rule denotes the percentage of transactions in 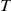 that contains both 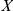 and 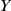. This is taken to be the probability 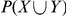. An association rule (AR) is called frequent if its support exceeds a minimum value 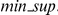


The confidence of a rule 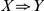 in 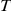 denotes the percentage of the transactions in 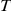 containing 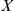 that also contains 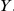 It is taken to be the conditional probability 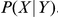 In other words,
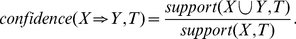
(5)A rule is called *confident* if its confidence value exceeds a threshold 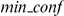. Formally the ARM problem can be defined as follows: Find the set of all rules 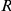 of the form 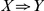 such that



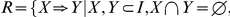


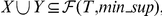


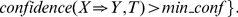
(6)Generally the ARM process consists of the following two steps [32], [33]:

1. Find all frequent itemsets.
2. Generate strong ARs from the frequent itemsets.

The number of itemsets grows exponentially with the number of items 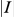. A commonly used algorithm for generating frequent itemsets is the *Apriori* algorithm [18], [34]. This is based on the concept of downward closure property which states that if even one subset of an itemset 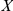 is not frequent, then 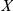 cannot be frequent. It starts from all itemsets of size one, and proceeds in a recursive fashion. If any itemset 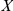 is not frequent then that branch of the tree is pruned, since any possible superset of 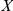 can never be frequent.

Although *Apriori* is a popular algorithm, its computational complexity becomes intractable for very low value of 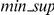 and when the number of items is very large. It is because low 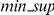 value generates very large number of frequent itemsets and generation of such a large number of frequent itemsets takes huge time. Moreover, it is necessary to ignore the redundant information in the frequent itemsets. In this context, the concept of *closed itemsets* [35], [36] is important. An itemset is called closed itemset if none of its proper supersets have the same support value. It is beneficial to search for the closed itemsets in order to avoid any redundancy. Moreover, frequent closed itemsets are condensed representation of frequent itemsets without loss of any information. There is no provision in Apriori or similar algorithms to directly search for the closed itemsets. In this article, we have proposed a novel approach that utilizes a biclustering method [37] for identifying the frequent closed itemsets directly.

The PPI data set considered in this article is very sparse (only 2534 interacting pairs among 27208 possible protein pairs: 9.31%). Therefore it is required to use low 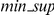 value for generating frequent itemsets. Moreover when the PPI matrix is arranged in a form in which the human proteins are considered as the items, the computational time required to execute the *Apriori* method is huge since the number of human proteins is 1432 in the data set considered here. This is because the number of possible itemsets to explore is in the order of 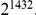 Furthermore, it is also important to find the frequent closed itemsets only to avoid redundant information. Therefore, it is difficult to use *Apriori* algorithm for this data set. Hence, we have proposed a new association rule mining approach based on a biclustering method that efficiently mines the frequent closed itemsets from sparse data sets having low 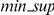 value and large number of items. The following section describes the proposed technique.

### Association Rule Mining based on Biclustering

In this section, we first introduce the concept of biclustering and thereafter, the proposed rule mining technique is described.

#### Biclustering

Biclustering technique, usually used in microarray gene expression data, aims to identify a subset of genes that are similarly expressed in a subset of experimental conditions [37], [38], [39]. Given a 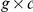 microarray data matrix 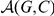 consisting of a set of 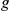 genes 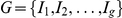 and a set of 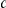 conditions 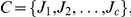 a bicluster can be defined as a submatrix 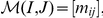 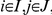 of matrix 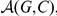 where 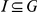 and 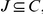 and the subset of genes in the bicluster are similarly expressed over the subset of conditions and vice versa. Biclustering algorithms have been mainly developed to overcome the shortcoming of standard clustering algorithms that fail to detect similarity of genes over a subset of experimental conditions [40], [41], [42].

Although biclustering algorithms are in general applied for microarray gene expression data sets, they can be used in any data matrix to discover submatrices with similar values. There are different kinds of biclusters available, viz., constant, row-constant, column-constant, additive pattern, multiplicative pattern, and combination of both additive and multiplicative patterns [37]. Constant biclusters are of special interest where all the elements of the biclusters have the same value. In case of a binary data set where all values are either 0 or 1, a constant bicluster can be thought as a submatrix, all the elements of which have value 1, while 0 is considered as the background value. Thus for binary data sets, identifying constant biclusters is equivalent to identifying all-1 submatrices from the data set.

#### ARM from All-1 Biclusters

Suppose a transaction data set is given that contains 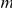 transactions and 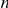 items. The data set can be represented as a binary matrix with rows representing transactions and columns representing items. If an element 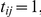 it implies that the item 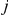 is purchased in transaction 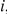 and if 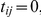 then the transaction 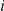 does not purchase the item 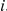 Given this binary matrix, if we can find an all-1 bicluster that has at least 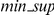 number of rows (transactions), then the set of columns (items) of that bicluster can be considered as a frequent itemset. Hence each all-1 bicluster that satisfies the 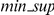 condition will provide a frequent itemset. Thus finding the set of frequent itemsets is equivalent to find a set of all-1 biclusters each having at least 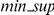 number of rows.

Among the various biclustering techniques available in literature, we have used Binary inclusion-Maximal (BiMax) biclustering algorithm [21] that identifies all biclusters in the input binary matrix. Based on this binary matrix, BiMax identifies all maximal biclusters where a bicluster is defined as a submatrix 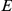 containing all 1s. An inclusion-maximal bicluster means that this bicluster is not completely contained in any other bicluster. Note that the columns of a maximal bicluster constitute a closed itemset. Hence the set of all-1 biclusters that satisfies the 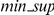 condition actually provides the set of frequent closed itemsets.

BiMax uses an incremental algorithm to find the inclusion-maximal biclusters exploiting the fact that the matrix 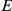 induces a biclique. An advantage of BiMax is that it does not need *a priori* specification of the number of biclusters to be found.

Therefore, to find the possible association rules, we use the following steps:

1. Given a 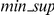 and a binary transaction matrix, apply BiMax algorithm with parameters minimum number of rows = 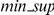 and minimum number of columns = 2 to find all frequent closed itemsets.
2. From each frequent closed 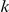-itemset, 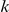 rules are generated by moving one of the items at a time to the consequent part while keeping the remaining items in the antecedent part of the rule.

Note that for generating the frequent closed itemsets, we ignore the 1-itemsets as they can not produce any association rule. This is done by fixing the minimum number of columns of the biclusters to 2. Moreover, we consider only the rules having single consequent to avoid redundant information, as our primary objective is to predict new PPIs.

## Supporting Information

File S1 **Excel file containing the 1432 × 19 PPI adjacency matrix with human proteins in rows and HIV-1 proteins in columns.**
(XLS)

File S2 **Excel file containing the 19 × 1432 PPI adjacency matrix with HIV-1 proteins in rows and human proteins in columns.**
(XLS)

File S3 **Excel file containing the interactions predicted from HV matrix, from VH matrix and their union.**
(XLS)

File S4 **Excel file containing intersection of the predicted interactions by the proposed method and that by Tastan et al.** [**11**] **.**
(XLS)
